# ADVANCING RESPITE RESEARCH TO SUPPORT FAMILY CAREGIVERS OF OLDER ADULTS

**DOI:** 10.1093/geroni/igae098.3372

**Published:** 2024-12-31

**Authors:** Kim Whitmore, Sarah Swanson

**Affiliations:** Marquette University, Sun Prairie, Wisconsin, United States; University of Nebraska Medical Center/Munroe-Meyer Institute, Omaha, Nebraska, United States

## Abstract

One of the most frequently expressed needs of family caregivers of older adults is respite – a temporary break from the responsibilities of caregiving. ARCH National Respite Network and Resource Center (ARCH) has advanced efforts to enhance the respite evidence base to assist with sustainability, promote continuous quality improvement, and help translate research into best practices. The Committee for Advancement of Respite Research (CARR) advises ARCH on the execution of its respite research initiative and is comprised of former members of the ARCH Expert Panel on Respite Research, research scholars and evaluators, and foundation representatives. This session will highlight the efforts of the CARR to address the following identified priority areas: • Define and measure the value (cost-effectiveness) of respite; • Recommend common data elements (CDE) for respite-related research; and • Expand culturally appropriate research with historically underrepresented populations This session will also provide an overview and discuss the application of the following products that have developed from the work of the CARR: • Measuring the Value of Respite which includes a proposed framework for research; • Recommended Common Data Elements (CDEs) for Respite-related Research; • Resources for Culturally and Linguistically Competent Respite Research; and • Ensuring Cultural and Linguistic Competence: A Guide for Respite Researchers. Finally, we will highlight how this work aligns with the research recommendations in the 2022 National Strategy to Support Family Caregivers.

